# Cognitive Behavioural Therapy for an Adolescent with Anorexia Nervosa

**DOI:** 10.3390/children9010092

**Published:** 2022-01-10

**Authors:** José I. Baile, María F. Rabito-Alcón

**Affiliations:** Department of Psychology, Open University of Madrid (UDIMA), 28400 Madrid, Spain; mariafrenzi.rabito@udima.es

**Keywords:** eating disorders, anorexia nervosa, cognitive behavioural therapy, psychological treatment, adolescence

## Abstract

Introduction: The treatment of anorexia nervosa remains a matter of much debate. Though cognitive behavioural therapy would seem to offer good results, there is still no resounding evidence pointing to a single treatment of choice. The case presented in this paper examines the treatment with CBT of a patient presenting anorexia nervosa. Evaluation/diagnosis: An adolescent girl, 17 years of age, voluntarily attends psychological therapy to address eating behaviour problems. After administering the EAT-26, EDI-2, and BSQ standardised screening tests, as well as a clinical interview for assessment, a psychopathological profile is obtained, providing a diagnosis of anorexia nervosa, restricting subtype. Therapeutic goals: The therapeutic goals set were to reach a healthy weight for the patient’s age and height (specified as a minimum BMI of 18.5) and change the structure of thoughts, feelings, and behaviour that was justifying and maintaining the disorder. Treatment: Treatment lasted for 33 sessions and used cognitive behavioural techniques, such as cognitive restructuring, response cost, and positive reinforcement, in addition to family intervention techniques. Nutrition therapy was also carried out in parallel to the treatment sessions. Results: Following eight months of weekly sessions, the patient reached the target weight and changed attitudes towards food and body image, replacing them with healthy thoughts and behaviours. Follow-up made one and two years after the end of the treatment saw that these results were maintained. Discussion and conclusions: In this case, CBT proved effective in achieving the patient’s physical and psychological recovery. Therefore, this case contributes to the evidence of the efficacy of this therapeutic approach in certain cases of ED.

## 1. Introduction

Eating disorders (EDs) remain a significant health problem in today’s society. Their incidence and prevalence remains high among adolescents [1] in Western countries, including Latin America [2], with similar rates. Symptoms among patients, both physical (weight loss, malnutrition, fatigue, and insomnia) and psychosocial (loss of social relationships, problems at school, and decreased sexual activity), tend to be severe [3].

Additionally, considering that anorexia nervosa has the highest mortality rate of all psychiatric illness [4], determining which treatments are most effective in treating patients is a matter of vital importance.

Cognitive behavioural therapy (CBT) has shown to be efficient in treating some types of eating disorders [5], such as bulimia nervosa and binge eating disorder, but there is less evidence of its efficiency in patients with anorexia nervosa [6]. Anorexia nervosa is characterised by a significant loss of weight and a pathological desire to be thin. It is a difficult disorder to treat, which commonly lasts several years and in which constant relapses occur in a high percentage of cases [7,8].

This paper examines the treatment of a case of anorexia nervosa in an adolescent girl with CBT, with the aim of contributing greater evidence of this therapy’s effectiveness in treating eating disorders.

## 2. Case Presentation

### 2.1. Therapeutical Context

The patient was treated at the Centro de Orientación y Atención Psicológica Integral (COAPI), part of the Department of Psychology of the Universidad del Valle de Atemajac in Guadalajara, Mexico. She was treated by a psychologist trained in cognitive behavioural therapy, as well as a nutrition specialist. The patient had initially sought nutritional counselling services for the purpose of improving her diet. This service, however, suspected the existence of a psychological illness and referred the patient to COAPI.

The treatment was carried out in keeping with the ethical and professional parameters of the university’s Department of Psychology and under the supervision of the director of COAPI. The patient provided written consent to publish this case anonymously.

### 2.2. Assessment

#### 2.2.1. Initial Exploration (First Session)

The patient attends the first interview with her mother, and they are invited to be present together in the first session. The patient is a 17-year-old girl who lives with family members. Her physical condition can be described as emaciated, with extreme thinness. There are no signs of disorientation, mental confusion, or of agitated thought or speech. The patient is aware she has a problem and that it is getting worse (she cannot get out of bed in the morning, experiences dizziness, and a worsening academic performance), but she does not consider it especially serious nor does she recognise it as anything specific, only calling it “problems with food”. However, she does acknowledge that she eats very little, some days having only two or three pieces of fruit. Until recently, she did a lot of physical exercise (aerobics at a gym several times a week in addition to exercise at home), but now these routines have been greatly reduced due to her lack of strength. She has not menstruated in seven months. As background, she explains that two years ago she was overweight and that her problems began when her friends would make fun of her appearance. Therefore, we can estimate that the disorder began when she was 15 years old.

Part of the interview explores whether the patient remembers any episodes of trauma from her childhood; no significant findings are obtained with regard to this aspect, which could be an indicator of a good prognosis. The patient’s academic performance has always been very good and she has always received high marks, indicating a habitual perfectionist personality trait. She is worried about her marks going down, as recently she has missed some days of school due to fatigue or dizziness.

The patient does not believe she is in bad health and, initially, does not seem to have much interest in making changes. She is certain that she wants to remain thin, no matter what.

She has brought with her a doctor’s report that makes a generic reference to eating disorders and to slight anaemia, but without further complications of any other kind. Her doctor continued performing medical follow-ups throughout the period of psychological treatment to ensure there were no complications requiring the use of another type of treatment (medical or psychiatric).

The patient’s weight and height are recorded (this is performed in every session): 37 kg (81.6 pounds), 1.57 m (5 feet 1.8 inches), and a body mass index (BMI) of 15.04 kg/m^2^. This indicates that the patient is dangerously thin, according to the World Health Organisation’s international parameters for interpreting BMI.

A preliminary diagnostic hypothesis of anorexia nervosa is reached.

#### 2.2.2. Specific Assessment

A more specific assessment is carried out during the second and third sessions, with two objectives: (1) to understand the characteristics of the disorder affecting this patient in particular, and thereby determine the treatment; and (2) to establish a diagnosis.

In the second session, an assessment was carried out using versions of the following instruments validated for Spanish speakers:

- Interview for Diagnosis of Eating Disorders (IDED) [9] (Williamson 1990), to explore whether the characteristic signs and symptoms of ED are met;
- Body Shape Questionnaire (BSQ) [10], to assess the degree of body dissatisfaction. The score obtained in the first session was 176 points; the most appropriate cut-off point for the Mexican population is estimated to be 110 points [11];
- Eating Attitudes Test-26 (EAT-26) [12], to assess the risk of suffering an ED. The score obtained in the first session was 56 points; the most appropriate cut-off point for the Latin American population is estimated to be 22 points [13];
- Eating Disorder Inventory-2 (EDI-2) [14], to establish a psychopathological profile, in different variables, in patients with EDs. With this questionnaire a profile of different scales is obtained, and there is no established cut-off point.

As the patient does not present any other symptoms suggesting the presence of another disorder, the presence of comorbidity can be disregarded for the time being. The presence of comorbidity would require a specific assessment, and this can be reconsidered should symptomology compatible with another pathology appear in future sessions.

In the third session, the results obtained by the assessment instruments in the previous session are discussed with the patient, and evaluations are made by interview to:

- Explore the DSM diagnostic criteria for anorexia nervosa;
- Explore the patient’s family structure and its dynamics;
- Train the patient in keeping records of her eating behaviour and physical activity. Self-registers were designed in which the information about the place, time, planned meal, meal, thoughts, and emotions was indicated. Similar self-registers were used to record physical activity.

### 2.3. Case Conceptualisation

The patient is a 17-year-old adolescent presenting an eating disorder. The following results are obtained from the assessment:

- Psychometric tests point to passing all the cut-offs that indicate the risk of suffering from an ED, in addition to a clearly pathological profile in various EDI subscales, including a drive for thinness and body dissatisfaction;
- Information on the existence of thoughts, emotions, and behaviour characteristic of EDs has been obtained from the interview;
- All the DSM diagnostic criteria, restricting type, have been met;
- The patient displays a severely deteriorated physical condition, significant psychosocial alteration, and the family dynamics are strongly affected.

### 2.4. Goals of Treatment

- Treatment goals are divided into an overall goal and other specific goals;
- Overall goal: recovery of overall health;
- Specific goals:

1. Recovery of a state of physical health associated with regaining weight until reaching a minimum acceptable for the patient’s age, gender, and height (BMI between 18.5 and 21);

2. Recovery of psychological health, specified as: absence of an obsession with body image, the absence of a compulsion to engage in physical exercise, improvement in eating habits in terms of healthy amounts and qualities, and an improvement in self-acceptance of the patient’s body image.

The therapeutic recovery will be considered successful, once both specific goals have been achieved.

### 2.5. Treatment Techniques

The treatment plan established includes weekly sessions of psychological therapy with a cognitive–behavioural focus (following the main indications of ED treatment manuals [15], adapting them to the case), lasting one hour per session plus homework assignments; and another, 30 min weekly session with a nutrition specialist. The patient attends the sessions by herself, except for certain cases for which session work is performed with the mother or other members of the family. Therapy is organised into the following three phases.

#### 2.5.1. PHASE 1. Objective: Getting the Patient above the Risk Threshold

Techniques used:

1. Training in calculating BMI. First, the therapist and patient reach an agreement on what the target BMI should be. Next comes the design of a self-administered assessment scale of the patient’s personal situation according to her BMI at any moment. Lastly, a goal is set for the therapy agreed upon: “being thin, but healthy”;

2. Training in keeping records and self-observation. Keeping records of the situational, cognitive, emotional, and behavioural aspects related to food behaviour. Recording basic details of what, when, how, and under what circumstances;

3. Behavioural analysis, ABC analysis technique, detection of cognitive errors justifying altered behaviours, mainly cognitive distortions;

4. Establishment of food behaviour guidelines, mainly:

- Eat with someone else;
- Start by eating “everything”, even if in small quantities;
- Perform muscle relaxation exercises after eating;
- Positive self-talk while eating or immediately afterwards to counteract obsessive and paralysing thoughts.

5. Reinforcement of the goals achieved (increases in BMI, reduction in pathological behaviours, and increase in healthy behaviours) during sessions with the therapist and through enjoyable and meaningful activities;

6. Stopping improper behaviours related to food.

#### 2.5.2. PHASE 2. Objective: Consolidate Achievements, Modify Patterns More Permanently (15 Sessions)

Techniques used:

(a) Cognitive and emotional restructuring.

The following strategies are used to undo the distorted emotional–cognitive style maintaining the eating disorder:

- Development of the acceptance of body image through cognitive restructuring and psychoeducation;
- Undoing the ideas that justify pathological behaviour to achieve thinness by means of the Socratic dialogue and cognitive restructuring;
- Use of the Socratic dialogue to disassociate the value of a person and success in life from being thin;
- Knowing how to identify and control the pattern of thought > emotion > behaviour > reinforcement;
- Using the technique of thought-stopping to reduce obsessive thoughts.

(b) Behaviour modification.

- Utilising the social reinforcement of achievements that can include eating the amount planned for the day or going for a certain amount of time without excessive exercise;
- Stopping undesirable behaviours, e.g., ignoring constant complaints of stomach pain after eating the planned amount of food;
- Material reinforcement, in this case tokens were awarded to be later turned in for a weekend trip as a prize;
- Training in problem-solving to deal with difficulties at school and with classmates;
- Social skills training to overcome the skills deficits detected in the relationships with family members and at school;

(c) Family intervention.

- Motivational interview with each of the closest family members: mother, grandmother, aunt, and sister;
- Detection of family problems that interact with the disorder, especially concerning the lack of common goals, the family not understanding that the adolescent is suffering from a serious illness, and the tendency to solve problems through pressure and arguments.
- Guidelines for family intervention: remove the element of conflict from the illness, dispense with all the accusations and arguments; family arguments must not take place in front of the adolescent, especially during meals; progress must be encouraged instead of punishing any “lack of progress”; the common therapy goal must be clearly understood; and the family must collaborate in achieving it and participate in material reinforcement;
- Regular follow-up is made of whether family agreements are kept.

(d) Bibliotherapy on nutrition and the physiological effects of malnutrition using reading material selected specifically by the therapist: two books [16,17] published for Spanish-speaking populations were used as the base material.

#### 2.5.3. PHASE 3: Objective: Relapse Prevention (5 Sessions)

The process of this phase followed these steps:

(a) Development and application of a “therapeutic weaning” plan aimed at gradually reducing the intensity and frequency of therapy;
(b) Reinforcing the patient’s perception of recovery by her own means and efforts, using examples and revisiting achievements. Encouragement of self-management and self-regulation;
(c) Emphasising:
  - Positive aspects achieved with the new situation;
  - Which elements can work best to reinforce these achievements?
(d) Indicate the strategies to prevent relapse, including:
  - Examining which therapeutic techniques have proven most effective and establishing how they can remain effective;
  - Keeping a record of meals and behaviour for several months, or resuming record-keeping when alterations in eating behaviour are recognised;
  - Following a daily meal plan for several months without breaking it;
  - Learning to identify stimuli and situations that can provoke new negative thoughts and that can alter eating patterns, and how to either avoid them or implement diversionary or alternative behaviours;
  - Not stepping on the scale more than once a week or every 15 days;
  - Designating a person the patient trusts and confides in to speak with when having doubts regarding a possible relapse, in this case a friend;
  - Learning to recognise relapse in order to turn to the strategies learned, and knowing how to recognise when a situation has worsened in order to, once again, seek specialised help.

## 3. Results

The intervention lasted eight months. The patient’s weight was evaluated once a week, and progress in psychological variables was measured once every three weeks. By the end of therapy, the patient had gradually gained back 12 kg (26.5 pounds) and had resumed regular menstruation. The final results of the psychological tests showed that the patient was out of the pathological range, making it possible to end therapy.

**Figure 1:**
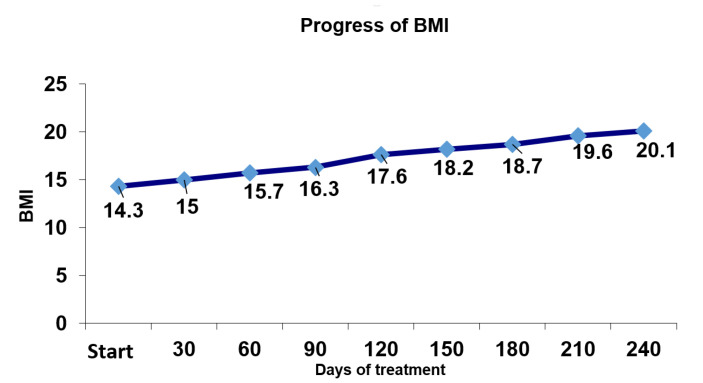
Progress of body mass index (BMI) over the course of therapy.

To analyse the results from the perspective of the proposed therapeutic goals, the progress of BMI was tracked over the course of therapy. Figure 1 shows that the goal of 20 kg/m^2^ was met, surpassing the established minimum of 18.5 kg/m^2^.

**Figure 2:**
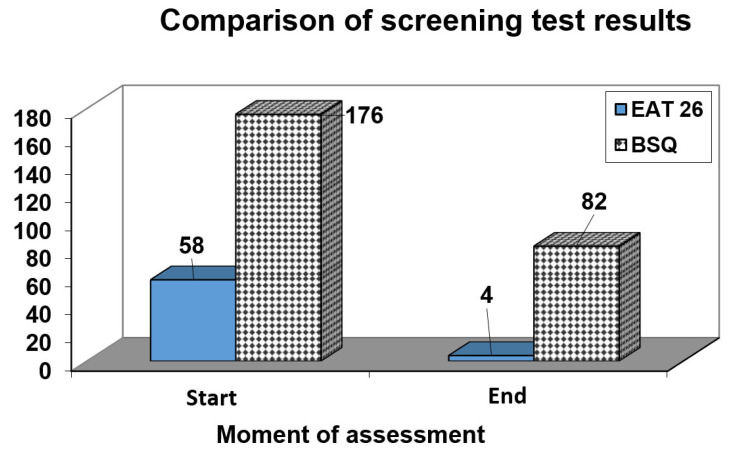
Comparison of EAT-26 and BSQ results between the beginning and end of therapy.

In the final sessions of treatment, during the relapse prevention phase, the patient retook the assessment questionnaires used in the initial assessment (EAT-26, BSQ, and EDI-2). In all three, the patient’s results were outside the pathological levels. Figure 2 compares two of the questionnaires between the beginning and end of therapy. On EDI-2, following treatment, the profile no longer showed the usual psychopathological format and all the subscales were below pathological levels.

Once therapy was concluded, the maintenance of results was followed up. At the initial follow-up interview, taking place at the COAPI one year after the end of therapy, an anthropometric assessment is made (BMI = 21.3 kg/m^2^) and signs of ED symptoms are checked for. Another follow-up takes place by telephone two years after the conclusion of therapy to check for ED symptoms. Both interviews confirmed that the positive results were being maintained and that the BMI had even improved (within healthy parameters, BMI = 21.5 kg/m^2^), in addition to the psychosocial variables.

## 4. Discussion and Conclusions

The cognitive–behavioural approach to therapy was effective in this case, as is seen in the results presented. The proposed goals were met within a relatively acceptable time frame, and the patient successfully recovered her health.

Several considerations must be put forth, which arose in the case and affected the therapy to some degree. For one, in some cases, the family’s socio-economic level made it difficult to set certain patterns of healthy eating and limited the use of material reinforcement. The elevated absence of a family structure also proved to be a determining factor and required individual intervention with each member of the family, as it was not possible to work via group therapy. Nevertheless, all of the family members were clearly worried about the situation and offered their collaboration in reaching the therapy goals.

As positive considerations, the patient was highly motivated to change and developed a growing concern with improving her physical appearance as well as her social and sentimental relationships. It would seem that the recovery of the patient’s physical condition was accompanied by the resumption of menses and other physiological changes related to puberty, which had been delayed for several years due to the extreme thinness and that had been a cause for great concern to the patient.

- It is important to note that two factors were present, which singularly favoured the success of the treatment:
- The awareness of the existence of a health problem. Although the patient did not think she was suffering from an ED, the presence of dizziness and the worsening academic performance worried her enough to create a starting motivation for change, which paved the way to establishing a therapeutic relationship and making a commitment to change;
- The positive initial results of some of the techniques used gave the patient a high degree of confidence in the therapy and bolstered her commitment. For example, though the first meals had caused much physical discomfort, the use of muscle relaxation techniques following meals proved extremely useful in resolving one of the main excuses for not continuing with the proposed meal plan.

The primarily behavioural and cognitive techniques used were supplemented with more familiar strategies, which, it is believed, were essential to the success achieved. The strategy followed was one commonly recommended for a cognitive–behavioural approach [18], beginning with the more behaviour-related techniques to establish eating habits and recover weight, and afterwards applying the cognitive techniques to disarm the pathological cognitive style and replace it with a more adaptive and healthy style. Therefore, it can be concluded that the usual procedure is adequate, at least as seen in this case.

Therefore, it can be concluded that, in this case, the cognitive–behavioural approach was successful in treating a case of anorexia nervosa, which is consistent with other findings in different areas, such as hospitals [19]. However, this approach has not always received the same support in scientific literature [6], despite it also being true that attempts have been made to discover enriched variations of this therapy [20] in pursuit of a more efficient type of therapy. This has been the case with enhanced cognitive behaviour therapy, of which insufficient studies exist at present to determine its efficacy, though the results obtained thus far have shown promise [21].

## Data Availability

Not applicable.
